# Using radiomic features of lumbar spine CT images to differentiate osteoporosis from normal bone density

**DOI:** 10.1186/s12891-022-05309-6

**Published:** 2022-04-08

**Authors:** Zhihao Xue, Jiayu Huo, Xiaojiang Sun, Xuzhou Sun, Song tao Ai, LichiZhang, Chenglei Liu

**Affiliations:** 1 Institute for Medical Imaging Technology, School of Biomedical Engineering, Shanghai Jiao Tong University, Shanghai, China; 2 Shanghai Key Laboratory of Orthopaedic Implants, Department of Orthopaedic Surgery, Shanghai Ninth People’s Hospital, Shanghai Jiao Tong University School of Medicine, Shanghai, China; 3 Department of Radiology, Shanghai Ninth People’s Hospital, Tong University Shanghai Jiao School of Medicine, Shanghai, China

**Keywords:** CT, Lumbar spine, Osteoporosis, Radiomics

## Abstract

**Objective:**

This study aimed to develop a predictive model to detect osteoporosis using radiomic features from lumbar spine computed tomography (CT) images.

**Methods:**

A total of 133 patients were included in this retrospective study, 41 men and 92 women, with a mean age of 65.45 ± 9.82 years (range: 31–94 years); 53 had normal bone mineral density, 32 osteopenia, and 48 osteoporosis. For each patient, the L1–L4 vertebrae on the CT images were automatically segmented using SenseCare and defined as regions of interest (ROIs). In total, 1,197 radiomic features were extracted from these ROIs using *PyRadiomics*. The most significant features were selected using logistic regression and Pearson correlation coefficient matrices. Using these features, we constructed three linear classification models based on the random forest (RF), support vector machine (SVM), and K-nearest neighbor (KNN) algorithms, respectively. The training and test sets were repeatedly selected using fivefold cross-validation. The model performance was evaluated using the area under the receiver operator characteristic curve (AUC) and confusion matrix.

**Results:**

The classification model based on RF had the highest performance, with an AUC of 0.994 (95% confidence interval [CI]: 0.979–1.00) for differentiating normal BMD and osteoporosis, 0.866 (95% CI: 0.779–0.954) for osteopenia versus osteoporosis, and 0.940 (95% CI: 0.891–0.989) for normal BMD versus osteopenia.

**Conclusions:**

The excellent performance of this radiomic model indicates that lumbar spine CT images can effectively be used to identify osteoporosis and as a tool for opportunistic osteoporosis screening.

**Supplementary Information:**

The online version contains supplementary material available at 10.1186/s12891-022-05309-6.

## Introduction

Osteoporosis is a common age-related bone metabolic disorder characterized by bone mineral loss and reduced strength, leading to an increased risk of bone fractures [1]. With longer life expectancy, the incidence of osteoporosis and fragility fractures is increasing, especially among postmenopausal women, worsening their quality of life and posing a substantial socioeconomic burden on patients and society [2]. Patients are often not diagnosed with osteoporosis prior to osteoporotic fractures; thus, routine identification of at-risk patients is desirable. Furthermore, osteoporosis is considered a major cause of surgery instrumentation failure, including screw loosening and pull-out after spinal fusion surgery [3]. Therefore, knowledge of a patient’s bone mineral metabolism before surgery plays a critical role in reducing postoperative complications.

Currently, the most widely used method for diagnosing osteoporosis is based on the assessment of bone mineral density (BMD) using dual-energy X-ray absorptiometry (DXA) [4]. However, this method has inherent limitations [5]: DXA fails to accurately reflect the real BMD in patients with severe degenerative spine disease, spine deformity, aortic calcification, and obesity due to technical limitations. Furthermore, BMD alone is insufficient to determine bone strength. Characterization of the structural and physical properties of the trabecular bone is crucial for the assessment of bone quality and the identification of fracture risk [6]. Therefore, a clinically feasible tool is urgently needed to improve the diagnosis of osteoporosis in the spinal region.

Lumbar CT is routinely used in the diagnostic process of patients with low back pain. Previous studies have shown that the average density of the lumbar vertebral bodies measured in Hounsfield units (HU) is significantly associated with BMD [7]. The average HU value derived from the lumbar vertebrae can be used to detect osteoporosis or predict vertebral fractures [8, 9]. However, the CT values are influenced by different acquisition parameters, such as scanning voltage and manufacturer (e.g., GE, Philips, Siemens, Toshiba) [10]. In contrast to CT HU values, the radiomic features extracted from the CT vertebral images are insusceptible to varying image acquisition, reconstruction, segmentation, and data modeling [11]. This image processing approach is reproducible, repeatable, and robust and is expected to improve diagnostic accuracy.

Using the radiomic approach, imaging features extracted from the segmented regions may quantitatively reflect lesion heterogeneity [12]. Radiomics is commonly used in clinical oncology for cancer detection, diagnosis, prognosis, and treatment response prediction [13], while rarely employed to investigate bone diseases. Recently, several studies have reported using textural features extracted from x-ray/MRI/DXA for osteoporosis detection, diagnosis, and bone disorder classification [14–16]. However, the predicted performance for osteoporosis diagnosis is still insufficient, and the area under the curve (AUC) reported for osteoporosis classification is approximately 0.8. These results may be attributed to the fact that the selected region of interest (ROI) was not a 3D reconstruction of the whole vertebral body, consequently leading to the loss of some possibly relevant information and affecting the precision of the predictive model. Therefore, further research is necessary to improve predictive performance. Multi-detector CT images were converted into volumetric data, more suitable for 3D radiomic feature analysis of the trabecular bone. At present, reports on lumbar CT radiomics for osteoporosis classification are lacking. Therefore, the present study aimed to investigate the utility of radiomic analysis based on lumbar spine CT images in distinguishing osteoporosis from normal bone density.

## Materials and Methods

### Subjects

This retrospective study was approved by the institutional review board of the Shanghai Ninth People’s Hospital, and the need for informed consent was waived. Between January 2019 and August 2020, 553 patients were admitted to the hospital for low back pain and were retrospectively reviewed for inclusion. Among them, 260 patients with available DXA reports and lumbar spine CT images were initially selected. The exclusion criteria were as follows: 1) previous lower lumbar surgery with metal fixation or bone cement (*n* = 80); 2) bone metastases (*n* = 6); 3) hematological disorders (*n* = 3); 4) metabolic bone diseases other than osteoporosis (*n* = 17); 5) poor DXA image quality (*n* = 2); and 6) L1-L4 vertebral body fracture or compression and deformation (*n* = 19). In total, 133 patients were included in this study.

### Image Acquisition and Preprocessing

All BMD examinations were performed using a DXA system (Discovery-A, Hologic Inc. Marlborough, MA, USA) with 140 kVp and 2.5 mAs. Prior to the examination, quality assurance procedures were routinely performed. BMD reports were based on the spine (L1–L4) and hips. Based on the WHO diagnostic criteria [4], the patients were divided into three groups: 1) normal BMD and patients with a T-score ≥  − 1.0 standard deviations (SD); 2) osteopenia: patients with a T-score between − 2.5 and − 1 SD; and 3) osteoporosis: patients with a T-score ≤  − 2.5 SD. CT acquisition and sagittal reformation of the lumbosacral spine of all patients were performed using a 64-slice spiral multidetector CT (Brilliance 64; Philips, Amsterdam, Netherlands).

**Fig. 1:**
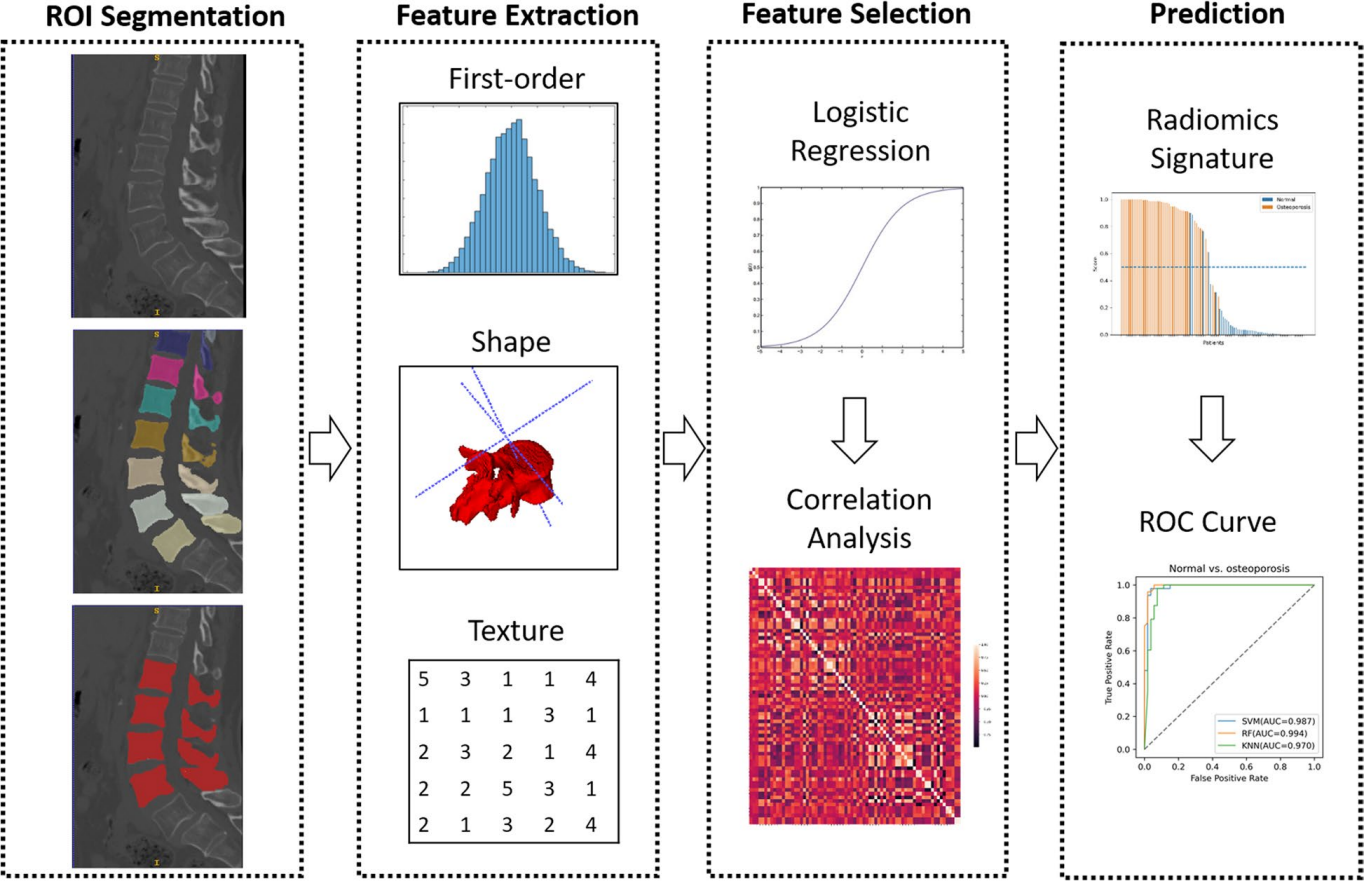
Image acquisition, processing, radiomic analysis, and modeling pipeline

For fully automated vertebral segmentation and identification from CT images, we used SenseCare [17], a research platform for medical image informatics and interactive 3D visualization. Next, the segmented images were reviewed, and the boundaries of the whole vertebral bodies were revised manually by a radiologist (CL) with eight years of experience, blinded to the BMD reports. Then, the L1–L4 vertebral bodies from each patient were selected as the ROIs for radiomic analysis. An example of ROI delineation with the overview of the framework is shown in Fig. 1.

### Preprocessing and Radiomic Feature Extraction

Radiomic features were extracted from the ROIs using *PyRadiomics*, an open-source package in Python that was recently developed for the automatic extraction of radiomic features from medical images [18]. Before the extraction, the image intensity was normalized; the 0.1 and 99.9 percentiles of each image were assigned as the minimum and maximum values, and values out of range were discarded. Then, the CT values were scaled to a range of 0–255 by using the following formula:

#### ${x}_{scored}=\frac{{x}_{raw}-min}{max-min}\bullet 255$,

to extract feasible features with *PyRadiomics*. In our study, we extracted 1,197 radiomic features in seven classes from each region (L1–L4): 1) first-order statistics (n = 234); 2) shape (n = 14); 3) gray level co-occurrence matrix (n = 286); 4) gray-level run-length matrix (n = 208); 5) gray level size zone matrix (n = 208); 6) neighboring gray-tone difference matrix (n = 65); and 7) gray level dependence matrix (n = 182), and several filters including Laplacian of Gaussian (LoG, σ = 1.0, 1.5, 2.0, and 2.5 mm) and wavelets. All the features extracted are listed in the Supplementary Data (Table S1).

### Radiomic Feature Selection

Since the radiomic features typically contain redundant information, a selection is required before constructing classification models. Before selecting them from the raw data, we scaled and zero-centered the features extracted using the Z-score method (i.e., ${x}_{scored}=\frac{x-\mu }{\sigma }$, where *μ* and *σ* are the mean and standard deviation of each feature). We built a logistic regression model with an L2 penalization term for feature selection[19]. If the corresponding coefficient of a feature was above the threshold (set as the mean of coefficients by default), the feature was considered important. The maximum number of features to select was empirically set to 150. Five-fold cross-validation was applied to determine the reliability of the selected features; those found in all five validation results were selected as the important features related to spine bone mass subtypes. All the feature selection methods above were applied to three types of binary classification tasks: normal BMD vs. osteoporosis, osteopenia vs. osteoporosis, and normal BMD vs. osteopenia.

### Classification Model Construction

We built three machine learning classifiers based on the feature selection results to divide the samples into positive and negative. Support vector machine (SVM), random forest (RF), and K-nearest neighbor (KNN) were chosen as the classification algorithms. The predictive performance of the classification models was validated on our dataset using fivefold cross-validation. We also quantified the performance of the models using the receiver operating characteristic (ROC) curve, and the AUC was calculated for the assessment. Additionally, we calculated the precision, recall, and F1 score as supplementary information for prediction efficiency. All classification models were implemented using the *scikit-learn* toolkit in Python [20].

### Statistical Analysis

Statistical analyses were performed using Python software. Differences in clinical data were analyzed using the chi-square test and one-way analysis of variance (ANOVA). The numeric values of radiomic features were compared between two different categories using the independent test or Mann–Whitney U test as appropriate. All tests were two-sided, and *P* < 0.05 was considered significant.

## Results

### Baseline Patient Characteristics

Among the 133 patients selected, 41 were men and 92 women, with a mean age of 65.45 ± 9.82 years (range: 31–94 years). The demographic and clinical data are summarized in Table 1. Among these patients, 53 had normal T-scores, 32 had osteopenia, and 48 had osteoporosis. There were no significant differences in sex, age, or body mass index between the three groups (All, *p* > 0.05;Table 1).

**Table 1:** Patients’ demographic and clinical characteristics in different categories

Variables	normal BMD (*n* = 53)	osteopenia (*n* = 32)	osteoporosis (*n* = 48)	P value
Gender				0.074
Male	22(41.5%)	9(28.1%)	10(20.8%)	
Female	31(58.5%)	23(71.9%)	38(79.2%)	
Age(years)	65.28 ± 12.50	63.16 ± 7.43	67.17 ± 9.82	0.200
BMI	25.28 ± 3.52	24.76 ± 3.72	24.09 ± 2.81	0.208

BMI: Body mass index

### Radiomic Feature Selection

With the logistic regression method, 1,197 × 4 radiomic feature values were ranked based on their patient subtype discriminating ability. Highly relevant features were selected as candidates for the SVM classification model and were then compared between different categories.

**Fig. 2:**
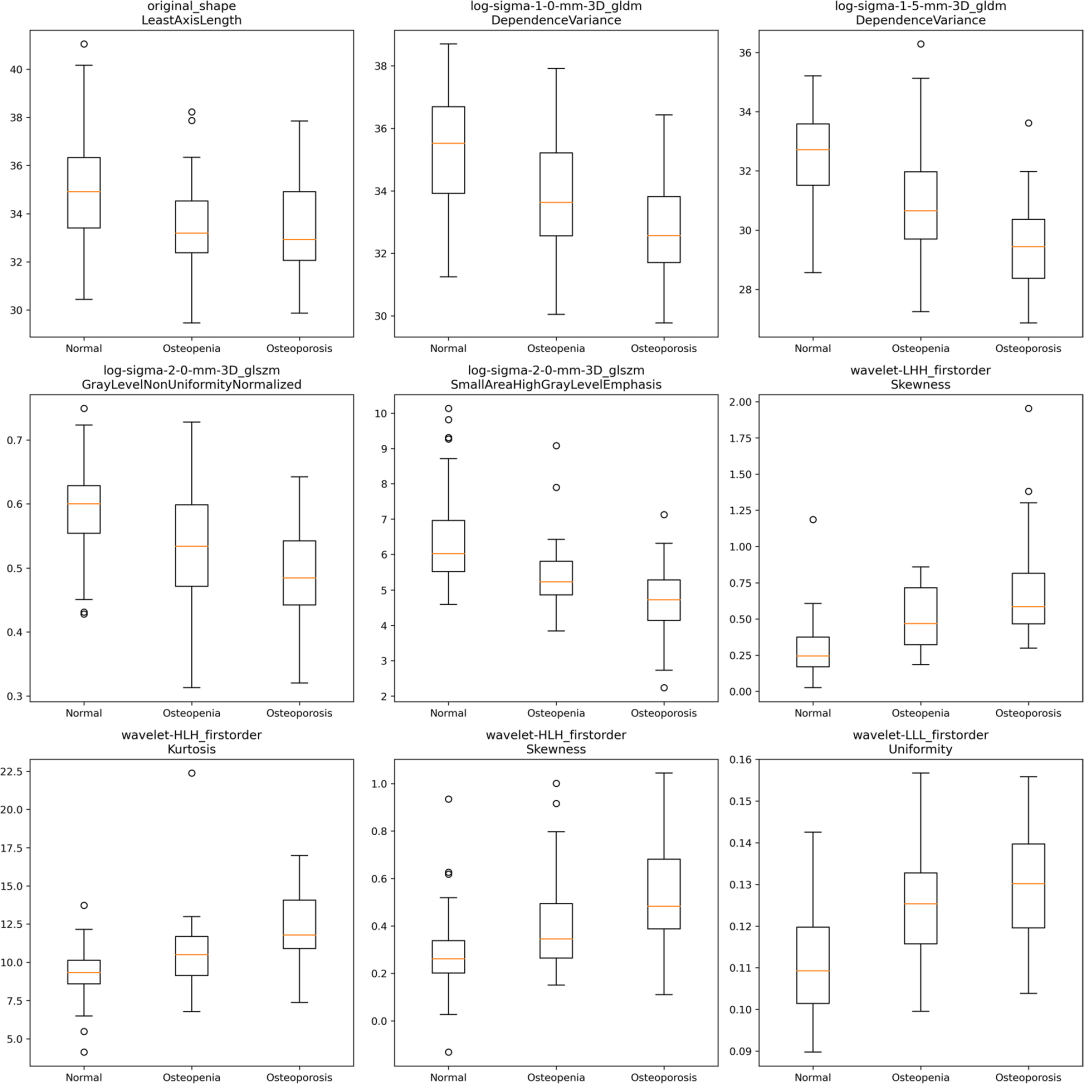
Comparison of a subset of selected L1-L4 radiomic features between different subtypes. The *x*-axis represents the different subtypes, and the *y*-axis shows the feature value

**Fig. 3:**
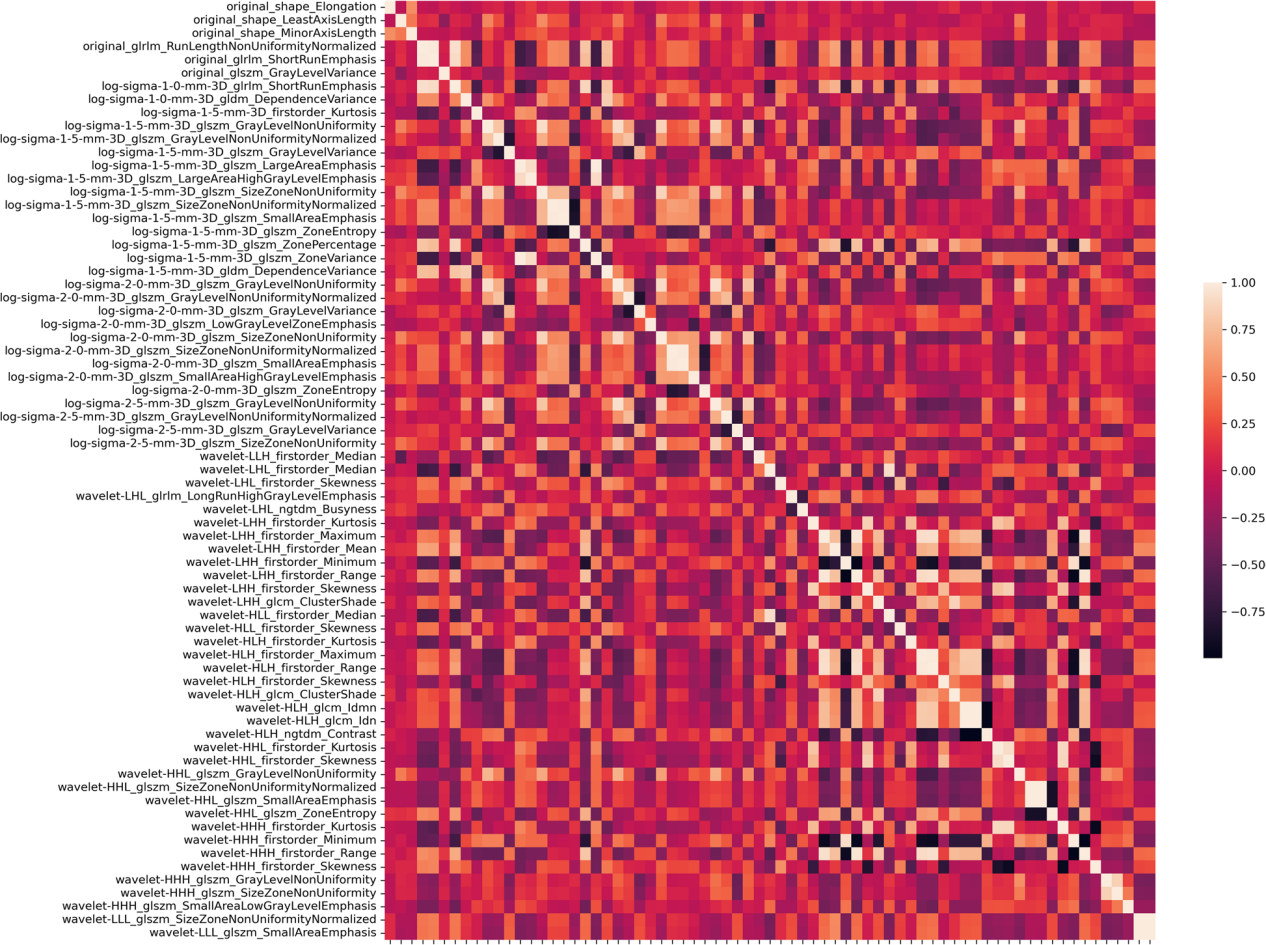
Each selected feature was compared with all other features, generating Pearson’s correlation coefficients (*r*). The *r* is shown as a heat map. A group of features with high correlation (*r* > 0.95) are redundant; thus, one feature should be chosen for the model, and the others can be omitted

For normal BMD vs. osteoporosis, 55 of the 4,788 features were selected as relevant; for osteopenia vs. osteoporosis, 20, and for normal BMD vs. osteopenia, 25. Nine typical features (one-way ANOVA: *P* < 0.05) are presented in the box plots in Fig. 2. For potential multicollinearity, we calculated the Pearson’s correlation coefficient matrix of the radiomic features (shown in Fig. 3). We eliminated 10 redundant features for the normal vs. osteoporosis comparison with the correlation matrix and statistical significance and chose the remaining 45 as highly representative. For osteopenia vs. osteoporosis, we preserved 19 of the selected features, and for normal BMD vs. osteopenia, 23 were finally selected. The differences in data distribution showed variations in feature values between the three subtypes. For instance, first-order statistics skewness, kurtosis, and uniformity in wavelet-filtered images were higher in subjects with osteopenia than in normal subjects. In contrast, texture features, such as the dependence variance of the gray-level difference method in Laplacian of Gaussian (LoG)-filtered images, were lower in subjects with osteoporosis and osteopenia.

### Model Construction and Performance Evaluation

**Fig. 4:**
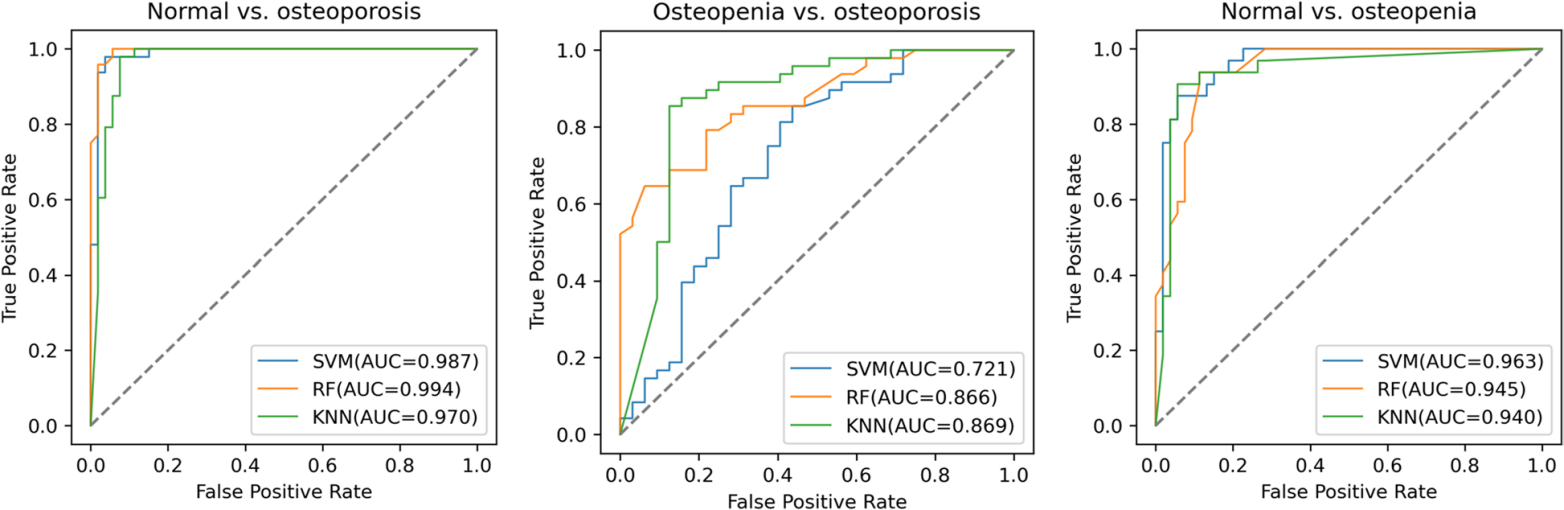
The receiver operating characteristic curve for the three classification tasks: normal vs. osteoporosis, osteopenia vs. osteoporosis, and normal vs. osteopenia

**Fig. 5:**
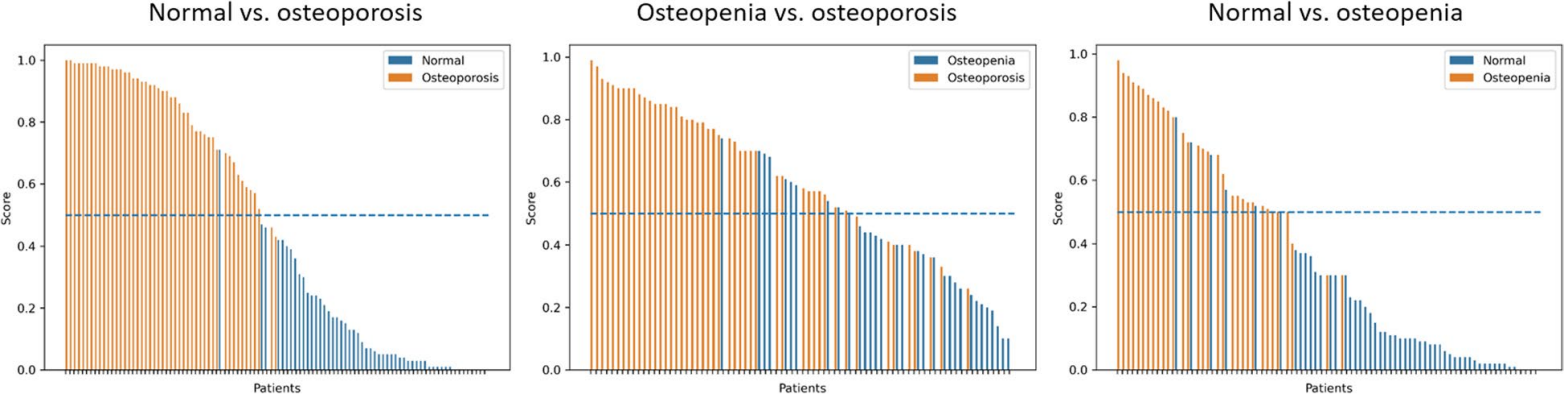
Bar charts of the prediction scores for each patient in the three tasks. The radiomic score in the *y*-axis represents the probability to be classified as positive by the support vector machine model. Each patient with a score above > 0.5 (blue dash lines) is classified as positive. Orange bars indicate true positives, and blue bars indicate true negatives

After selecting the optimal features, the RF, SVM, and KNN models were constructed to classify the samples in the dataset. The results indicated that the classification model based on RF had an excellent performance, with an AUC of 0.994 (95% confidence interval [CI]: 0.979–1.00) for differentiating normal BMD from osteoporosis, 0.866 (95% CI: 0.779–0.954) for differentiating osteopenia from osteoporosis, and 0.940 (95% CI: 0.891–0.989) for differentiating normal BMD from osteopenia. The ROC curves obtained for the three classification tasks are shown in Fig. 4. The AUCs obtained from the other two machine learning algorithms (SVM and KNN) and the confusion matrices for the predictions are presented in Table 2. The F1 scores were 0.970, 0.787, and 0.870 in the normal vs. osteoporosis, normal vs. osteopenia, and osteopenia vs. osteoporosis models, respectively. The waterfall plots in Fig. 5 show the relevance of the radiomic score to the classification categories.

**Table 2:** Confusion matrix, precision, recall, accuracy and F1 score of the predictions

		normal vs. osteoporosis		osteopenia vs. osteoporosis		normal vs. osteopenia	
		PN	PP	PN	PP	PN	PP
**Support vector machine**							
Confusion matrix	TN	51	2	17	15	48	5
	TP	1	47	7	41	4	28
AUC (95%CI)		0.987(0.964–1.00)		0.721(0.604–0.839)		0.962(0.924–1.00)	
Precision		0.959		0.732		0.848	
Recall		0.979		0.854		0.875	
Accuracy		0.970		0.725		0.894	
F1 score		0.970		0.716		0.894	
**Random forest**							
Confusion matrix	TN	52	1	23	9	48	5
	TP	2	46	8	40	6	26
AUC (95%CI)		0.994(0.979–1.00)		0.866(0.779–0.954)		0.945(0.899–0.992)	
Precision		0.979		0.816		0.839	
Recall		0.958		0.833		0.812	
Accuracy		0.970		0.788		0.870	
F1 score		0.970		0.787		0.870	
**K-nearest neighbor**							
Confusion matrix	TN	48	5	18	14	50	3
	TP	1	47	3	45	5	27
AUC (95%CI)		0.970(0.936–1.00)		0.869(0.783–0.956)		0.940(0.891–0.989)	
Precision		0.904		0.763		0.900	
Recall		0.979		0.938		0.844	
Accuracy		0.940		0.788		0.906	
F1 score		0.941		0.776		0.905	

TP, true positive; TN, true negative; PP, predicted positive; PN, predicted negative; CI, confidence interval

## Discussion

In the present study, we developed a predictive model for bone mineral loss using radiomic features extracted from CT images of the L1–L4 vertebral bodies. Our results revealed that the AUCs for the classification of normal BMD vs. osteoporosis, osteopenia vs. osteoporosis, and normal BMD vs. osteopenia were 0.994, 0.866, and 0.940, respectively, for the RF-based model. This high discriminatory power indicates that the radiomic features from lumbar spine CT images can be used as a new biomarker to predict osteopenia and osteoporosis. For patients who undergo lumbar CT, the radiomic features extracted from L1–L4 could be used as a tool for opportunistic osteoporosis screening. This image processing method is simple to implement and cost-effective, poses no additional radiation hazard, and may serve as a substitute for DXA.

In our study, the radiomic features extracted from lumbar spine CT images, including first-order and higher-order texture features, correlated with changes in BMD. A large subset of radiomic features was obtained from images filtered with an LoG operator and wavelet transform; these filtering methods can uncover image information invisible to the naked eye [21]. The LoG operation enhances intensity changes in CT images, and features extracted from processed images can more accurately reflect the structural differences of the vertebral bodies. Wavelet features contain high-order image information, comprehensively reflecting the spatial heterogeneity of the vertebral bodies.

Our results showed that the osteopenia and osteoporosis groups had higher skewness and kurtosis for multiple types of wavelet features. Kurtosis and skewness derived from histograms are two important parameters that indicate microstructural heterogeneity of the vertebral bodies. Kurtosis refers to the peakedness of the grey-level intensity distribution, whereas skewness represents the asymmetry. Higher kurtosis and skewness values reflect the increased complexity and heterogeneity of the selected ROIs [22, 23]. In this study, L1-L4 had higher kurtosis and skewness in patients with osteopenia or osteoporosis; this result may be attributed to increased bone marrow fat or changes in the microstructure of the trabecular bone. This finding is consistent with previous studies, which reported that patients with osteoporosis have more yellow fat in the vertebral bodies [24, 25]. However, this hypothesis needs to be confirmed in future pathological assessments.

Osteoporosis is characterized by a loss of bone strength and microarchitectural deterioration of the bone tissue [21]. Although BMD assessment using DXA indicates bone strength, it does not directly quantify microarchitectural information. The CT radiomic features of the vertebral bodies may provide meaningful and complementary information to assess bone quality. Therefore, models based on radiomic features extracted from CT images may reflect the underlying osteoporotic pathology more accurately. Using volumetric CT images and advanced imaging processing methods, we achieved higher predictive performance than a previous study for differentiating osteoporosis from normal BMD [14–16]. Our results showed an AUC of 0.994 to distinguish normal BMD and osteoporosis.

In comparison, Lee et al*.* developed a predictive model for BMD based on X-ray images; however, their highest performance was only approximately 0.7 [16]. Rastegar et al. explored the differentiation of osteoporosis and osteopenia from normal bone quality based on DXA images using a machine-learning radiomic approach, which yielded the highest performance (0.76) [14]. He et al*.* recently used a radiomic model based on lumbar MR images to detect osteoporosis; the AUC was approximately 0.79 [15]. We included the entire L1–L4 vertebral region, with each vertebra as an ROI; this method contributed to developing a more accurate predictive model. We also performed fully automatic vertebral segmentation and identification using an iterative fully convolutional neural network and cascaded 3D convolution networks [26, 27]. Automatic image segmentation can reduce the subjective variations typical of manual delineation.

Quantitative CT can be used to measure volumetric trabecular BMD, which is more sensitive and precise than DXA for detecting bone mineral loss by avoiding the superimposition of cortical bone and other soft tissues [28]. However, quantitative CT is rarely used in clinical practice because it requires specialized equipment and measurement software. In contrast, lumbar CT is routinely performed for the preoperative assessment of patients with low back pain. Opportunistic osteoporosis screening from lumbar CT imaging data can provide valuable preoperative information for spine surgeons since osteoporosis is a major cause of instrumentation failure after spinal fusion surgery [3]. Our predictive model with high discriminative power provides the possibility of opportunistic osteoporosis screening in non-dedicated lumbar CT scans. Based on the performance in our study, it can be expected that quantitative CT radiomic features can be regarded as a reliable substitute for DXA in the preoperative setting

Our study has some limitations. First, it was conducted in a single center, and the sample size was limited. Further research is needed on larger, multi-center CT imaging datasets. Second, we used L1–L4 as ROIs and analyzed the model performance based on all features without comparing different models for a specific vertebral body. Those comparisons may demonstrate the advantage of multi-regional radiomic models compared to single-region ones; further investigations on this aspect are needed in future studies. In addition, we only used the features extracted from CT images to rank the scores for osteopenia and osteoporosis. Including clinical risk factors (e.g., age, sex, and BMI) may also help improve the model’s performance as a predictor of bone mineral loss

In conclusion, our machine learning model based on 3D radiomic features from lumbar spine CT images can accurately differentiate bone mineral loss from normal bone density and be used for opportunistic osteoporosis screening

## Ethics approval and consent to participate

All procedures and methods in this retrospective study were carried out in accordance with the 1964 Helsinki declaration and its later amendments. The study design was approved by the institutional review board of Shanghai Ninth People’s Hospital, and informed consent was obtained from all individual particpants included in this study

## Supplementary Information

Below is the link to the electronic supplementary material.

Supplementary file1 (DOCX 16 kb)

## Data Availability

The data in this study are available on request from the corresponding author. The data are not publicly available due to privacy or ethical restrictions
