# Supplementary material for: Using radiomic features of lumbar spine CT images to differentiate osteoporosis from normal bone density
Source: BMC Musculoskelet Disord. 2022 Apr 8;23:336. doi: 10.1186/s12891-022-05309-6 (PMC8991484; doi:10.1186/s12891-022-05309-6)
Supplement: Supplementary file 1 — Supplementary file1 (DOCX 16 kb) [file 12891_2022_5309_MOESM1_ESM.docx]

**Supplementary**

Table S1. Extracted radiomics features

| First order | Shape | GLCM | GLRLM | GLSZM | NGTDM | GLDM |
| --- | --- | --- | --- | --- | --- | --- |
| 10Percentile | Elongation | Autocorrelation | Gray Level Non-Uniformity | Gray Level Non-Uniformity | Busyness | Dependence Entropy |
| 90Percentile | Flatness | Joint Average | Gray Level Non-Uniformity Normalized | Gray Level Non-Uniformity Normalized | Coarseness | Dependence Non-Uniformity |
| Energy | Least Axis Length | Cluster Prominence | Gray Level Variance | Gray Level Variance | Complexity | Dependence Non-Uniformity Normalized |
| Entropy | Major Axis Length | Cluster Shade | High Gray Level Run Emphasis | High Gray Level Zone Emphasis | Contrast | Dependence Variance |
| Interquartile Range | Maximum 2D Diameter Column | Cluster Tendency | Long Run Emphasis | Large Area Emphasis | Strength | Gray Level Non-Uniformity |
| Kurtosis | Maximum 2D Diameter Row | Contrast | Long Run High Gray Level Emphasis | Large Area High Gray Level Emphasis |  | Gray Level Variance |
| Maximum | Maximum 2D Diameter SLice | Correlation | Long Run Low Gray Level Emphasis | Large Area Low Gray Level Emphasis |  | High Gray Level Emphasis |
| Mean Absolute Deviation | Maximum 3D Diameter | Difference Average | Low Gray Level Run Emphasis | Low Gray Level Zone Emphasis |  | Large Dependence Emphasis |
| Mean | Mesh Volume | Difference Entropy | Run Entropy | Size Zone Non-Uniformity |  | Large Dependence High Gray Level Emphasis |
| Median | Minor Axis Length | Difference Variance | Run Length Non-Uniformity | Size Zone Non-Uniformity Normalized |  | Large DependenceLow Gray Level Emphasis |
| Minimum | Sphericity | Joint Energy | Run Length Non-Uniformity Normalized | Small Area Emphasis |  | Low Gray Level Emphasis |
| Range | Surface Area | Joint Entropy | Run Percentage | Small Area High Gray Level Emphasis |  | Small Dependence Emphasis |
| Robust Mean Absolute Deviation | Surface Volume Ration | Imc1 | Run Variance | Small Area Low Gray Level Emphasis |  | Small Dependence High Gray Level Emphasis |
| Root Mean Squared | Voxel Volume | Imc2 | Short Run Emphasis | Zone Entropy |  | Small Dependence Low Gray Level Emphasis |
| Skewness |  | Idm | Short Run High Gray Level Emphasis | Zone Percentage |  |  |
| Total Energy |  | Idmn | Short Run Low Gray Level Emphasis | Zone Variance |  |  |
| Uniformity |  | Id |  |  |  |  |
| Variance |  | Idn |  |  |  |  |
|  |  | Inverse Variance |  |  |  |  |
|  |  | Maximum Probability |  |  |  |  |
|  |  | Sum Entropy |  |  |  |  |
|  |  | Sum Squares |  |  |  |  |

GLCM, gray level co-occurrence matrix; GLRLM, gray level run length matrix; GLSZM, gray level size zone matrix; NGTDM, neighboring gray-tone difference matrix; GLDM, gray level dependence matrix
